# An overview of available drugs for management of opioid abuse during pregnancy

**DOI:** 10.1186/s40748-017-0044-2

**Published:** 2017-02-10

**Authors:** Jillian Laslo, Jon-Michael Brunner, Daniel Burns, Emily Butler, Autumn Cunningham, Ryan Killpack, Courtney Pyeritz, Kimberly Rinard, Jennifer Childers, Joseph Horzempa

**Affiliations:** 1 Department of Graduate Health Sciences, West Liberty University, West Liberty, WV USA; 2 Department of Natural Sciences and Mathematics, West Liberty University, West Liberty, WV USA

**Keywords:** Neonatal, Opioid, Abuse, Methadone, Buprenorphine, Buprenorphine/naloxone, Prenatal, Treatment, Pregnancy, Addiction

## Abstract

The prevalence of opioid abuse in the United States has been steadily increasing over the last several years among many major demographics, including pregnant women. Rise in prenatal opioid abuse has resulted in subsequent escalation of neonatal abstinence syndrome incidence, prompting the US Congress to pass the Protecting Our Infants Act of 2015. This act specifically calls for a critical review of current treatment options for prenatal opioid abuse which may ultimately lead to the development of better therapies and a decreased incidence of neonatal abstinence syndrome. Currently, the American College of Obstetricians and Gynecologists recommends methadone, buprenorphine, or buprenorphine/naloxone in the treatment of prenatal opioid abuse. In this review, each maintenance therapy treatment option is discussed and compared revealing inconsistencies in postpartum retention rates, effects on fetal development, and availability to patients due to restrictions in health care coverage. Although each of these treatment options reduces opioid abuse and potential negative outcomes for the fetus, the shortcomings of these drugs highlight the overarching need for an improved standard of care. Drug developers and lawmakers should consider that affordability, coverage by health insurance, and success in retention rates substantially impacts the decision of the patient and healthcare provider regarding utilization of a particular opioid maintenance therapy.

## Background

Opioid use in the United States has been steadily increasing since 2000 [1]. Between 2007 and 2013 alone, the number of users increased from 373,000 to 681,000 [1]. In 2014, 49.2% (18,893) of all drug abuse deaths in the United States were due to opioid abuse [2]. As opioid abuse continues to increase in the United States, one specific population at risk is pregnant women and their unborn fetuses. Among women that abuse opioids, early signs of pregnancy such as nausea, vomiting, and cramping are often confused with opioid withdrawal symptoms which leads to prolonged and sometime increased use of opioids. Because of the risks associated with these withdrawal symptoms, it is currently recommended for women addicted to opioids while pregnant to enter a treatment program consisting of methadone or buprenorphine [3].

Increases in drug use during pregnancy have resulted in an escalation in the occurrence of Neonatal Abstinence Syndrome (NAS), a drug-withdrawal syndrome that manifests after opioid penetration of the placental barrier resulting in-utero exposure [4]. The most dramatic increase was observed from 2009 to 2012, where NAS incidence increased nationally from 34 to 58 per 1,000 hospital births [5].

In addition to NAS, several other disorders and morbidities of neonates are associated with opioid use during pregnancy [6, 7]. For instance, infants who were subjected to opioids in utero have a higher incidence of stillbirth [6, 8, 9]. Moreover, use of particular prescription opioids during pregnancy is associated with congenital heart defects [10, 11]. The neonates who survive childbirth often experience a reduced growth rate compared to those whose mothers did not abuse opioids [6, 8, 9]. Secondary effects of opioid abuse often adversely affect the fetus as well. For example, women dependent on opioids have a higher incidence of anxiety and depression which may lead to aberrant neurological development of the fetus [6, 12, 13].

In response to the rise in prenatal opioid abuse and NAS, US Congress passed the Protecting Our Infants Act of 2015. This act requires the Department of Health and Human Services to evaluate and make recommendations on available treatments for women who continue to abuse opioids during their pregnancies, as well as identify any potential barriers of those existing treatments [14]. Currently, there are three drugs being used to treat general opioid dependence as well as opioid addiction among pregnant women; methadone, buprenorphine, and a combination drug containing buprenorphine/naloxone (B/N) [15]. The College of Obstetricians and Gynecologists recommends referral for opioid-assisted therapy with methadone, but also recognizes evidence in support of buprenorphine and B/N treatment creating a lack of consensus for a standard of care [16].

In an attempt to address the Protecting Our Infants Act of 2015, this paper will review strengths, weaknesses, and inconsistencies of the currently available treatment options for prenatal opioid abuse and address potential contributors to gaps in treatment research.

## Review of prenatal opioid abuse treatment options

### Methadone

Methadone is a synthetic opioid that has been commonly used for the treatment of opioid dependence and is primarily administered orally, sometimes intravenously, and rarely subcutaneously, spinally or through rectal routes. Structurally, methadone possesses a chiral carbon, and typically a racemic mixture of both the D- and L- isomers comprise the therapeutic form of this drug [17]. Either isomer binds to the μ-opioid receptors (acting as an agonist) and blocks the N-methyl-D-aspartate (NMDA) receptors (acting as an antagonist) thereby preventing monoaminergic reuptake transporters [17, 18]. Because methadone has both agonist and antagonist effects this drug leads to decreased tolerance compared to other opioids [17].

Methadone is a widely accepted treatment for opioid dependence among healthcare providers. Since the 1960s, methadone has been recommended as the standard of care showing no serious long-term side effects [19]. Methadone is well absorbed in the gastrointestinal (GI) tract and holds a long half-life of 25–52 h contributing to a low frequency of administration [17]. Methadone also has a stronger antagonist ability when binding to the NMDA receptors than the alternative therapeutics.

Methadone does have some serious drawbacks when considering administration. Because of opioid nature of this therapeutic and the longer half-life associated with methadone, there is potential for methadone overdose [20]. Moreover, methadone has been shown to cause harmful respiratory depression, constipation, and potential addiction and convulsions if toxic amounts are administered [17]. The US black box warning for methadone indicates that life-threatening QT prolongation and serious arrhythmias have occurred in patients using methadone. This warning urges patients to monitor for ECG changes during the start of treatment or after increasing the dose. In addition, utilization of methadone along with other central nervous system depressants such as benzodiazepines and alcohol can be fatal [21]. Furthermore, a fetus exposed to methadone is at risk for adverse perinatal outcomes such as decreased birthweight and NAS [22]. Unlike the other therapies discussed below, additional regulations regarding prescription methadone limit patient accessibility to this drug [23]. For instance, methadone is only available from healthcare providers that have been registered by the Drug Enforcement Agency as a substance abuse treatment facility. In these cases, the methadone must be administered by this registered facility. Moreover, methadone is not covered by Medicaid in many states, producing a financial burden that may further reduce accessibility of this drug to patients [23].

### Buprenorphine

Buprenorphine is a long-acting, potent phenanthrene derivative. This drug is often referred to as a mixed agonist–antagonist because buprenorphine acts as a partial agonist to μ receptors, and an antagonist to the δ and κ receptors, lessening the euphoria brought about by opioid abuse [17]. Buprenorphine is administered sublingually in order to avoid first-pass absorption. In addition to this prolonged absorption, buprenorphine also has a slow dissociation rate from μ receptors, which allows for a longer duration of action [17]. In addition, buprenorphine can also be administered as a subdermal implant

Pregnant women who use buprenorphine to treat opioid addiction have children with higher birth weights, larger head circumference, and fewer defects compared to those who use methadone [24]. Moreover, use of buprenorphine over methadone lowers the risk for preterm birth in pregnant women addicted to opiates [24]. In a retrospective cohort study that compared buprenorphine with methadone, mothers who used buprenorphine rather than methadone gave birth to larger infants and had longer gestation periods [19]. The infants of the buprenorphine group also required significantly less and shorter treatments for neonatal abstinence syndrome [19].

Buprenorphine, like methadone, has several disadvantages for mothers and infants. Both of these drugs may increase the risk of serious respiratory depression and death. This risk rises when buprenorphine is used in conjunction with benzodiazepines (psychoactive drugs used to treat anxiety), alcohol, patients who suffer from panic disorders, as well as central nervous system (CNS) depressants such as sedatives or antipsychotics [17]. Another drawback to using buprenorphine is that there is a lower retention rate for opioid dependence in patients who use buprenorphine (46%) versus methadone (74%) [24]. Moreover, buprenorphine-containing products are often abused, due to the ability of this drug to give a person a sense of euphoria when used in large quantities sublingually [25]. The potential for misuse of buprenorphine has led to increased illegal sales, sharing, and trading for other substances [25].

### Buprenorphine/naloxone (B/N)

The combination of B/N is used to treat opioid addiction and prevent buprenorphine abuse. As previously mentioned, buprenorphine is a partial agonist for the μ-opioid receptor. Buprenorphine’s agonist-binding activity blocks the receptor site, preventing opioids from attaching. Naloxone is a pure μ-opioid antagonist that not only binds to receptor sites to block opioid action, but can also reverse side effects of opioid use. Naloxone removes opioids from μ-receptors in the brain, which brings the individual into a state of withdrawal. The liver removes naloxone when taken orally during first-pass metabolism, therefore preventing transport to the brain. Because buprenorphine only partially binds the μ opioid receptor, the effects of this drug do not increase linearly with the dose and patients experience a “ceiling effect” as the activity of this drug plateaus above a certain concentration unlike the other opioid treatments. This ‘ceiling effect’ makes buprenorphine a safer choice than other full agonists at higher doses [26].

Of the current therapies to treat opioid addiction, perhaps the combination of B/N has the greatest potential. Because the pure opioid antagonist, naloxone, is included along with buprenorphine, there is minimal street value to this drug, and little likelihood for abuse. The withdrawal state experienced by the patient upon injection or intranasal administration further opposes misuse [25]. Generally, the retention rate of B/N increases with the dose of this treatment, and this therapy is associated with a high rate of completion (30–32 mg had a 24-week treatment plan completion rate of 60%) [27]. Patients taking B/N also exhibit a reduced relapse rate. For instance, approximately 55–80% of patients who use methadone relapse, and buprenorphine is found to have a 50% rate of relapse [28, 29]. In contrast, there are no relapses found in 79% of those treated with buprenorphine/naloxone [30]. Therefore, treating individuals with B/N significantly decreases the chance of opiate relapse [31]. Currently, women who become pregnant while taking B/N therapy are advised to discontinue use of naloxone, but maintain the buprenorphine regimen [16]. Because insufficient evidence exists regarding B/N utilization in pregnant women, there are potential risks associated with any undiscovered side effects or unfavorable outcomes.

The main shortcoming of utilizing B/N to treat opioid addiction is the cost. B/N is newer compared to other treatment methods for opioid abuse, making it more expensive than some of the other options while being manufactured under patent. Although the patent for B/N tablets expired in 2009 (making these currently available as a generic), Reckitt Benckiser introduced a new dosage form (a sublingual film strip) to the market which was filed under a new patent. Although other dosage forms of B/N exist, all remain more expensive in comparison to Methadone and Buprenorphine [31]. Because studies showed fewer adverse effects on the fetus when treated with B/N as compared to methadone and buprenorphine for opioid-dependence during pregnancy, this may be a superior treatment option [15]. However, cost, lack of coverage by health insurance, and an unwillingness to deviate from conventional treatments by some healthcare providers may impair the utilization of this drug.

## Discussion

Because of the inherent vulnerability of the developing fetus, obstetricians and neonatologists face many challenges associated with treating pregnant women who use opiates. Moreover, the existing maintenance therapies for opiate abuse have various shortcomings.

This review discusses three maintenance therapies for prenatal opioid abuse: methadone, buprenorphine, and B/N. Although methadone has been a standard of care for opioid abuse since the 1960s, utilization of this drug can have severe effects on an unborn fetus [19, 22]. Methadone is also not covered by Medicaid in several states, which presents a significant barrier in availability of this treatment option [23]. In contrast to methadone, utilizing buprenorphine in treatment of prenatal opioid abuse results in healthier babies born with higher birth weights and lower treatment times for NAS [19]. However, buprenorphine may still cause negative side effects in infants such as respiratory depression and has also been observed to have a lower postpartum retention rate in opioid dependent patients than methadone [17, 24]. B/N appears to present an improved treatment option in prenatal opioid abuse with 60% completion rate and decreased abuse likelihood due to lower street value [27]. However, the novelty of this treatment option limits its availability to patients and any potential benefits associated with its implementation [31]. For instance, being a newly developed drug, little evidence exists regarding the potential unrecognized dangers of B/N treatment during pregnancy. Future work should focus on determining whether the combinatorial B/N is safe to use during pregnancy.

Women who use opiates are often advised by their health care professionals to use contraception. These discussions are recommended to begin during pregnancy, and long-term but reversible contraception is often provided/prescribed after childbirth [16]. This is especially important as women who use drugs have a lower tendency to use planned (non-condom) contraception [32].

The aforementioned variability across prenatal opioid treatment effects and success may be accredited to demographic differences, such as race, ethnicity, socioeconomic status, and education level, and how those populations respond to accessible treatments. Research lacks focus on the effectiveness of methadone, buprenorphine, and B/N regarding specific demographic groups. The neglect of the impact of these demographic parameters in research studies – and how these parameters influence treatment availability and retention – may be contributing to the inadequate treatment success. Future studies should pay close attention to key demographic indicators, such as those mentioned above, that could influence healthcare decisions based on patient income or level of healthcare coverage.

## Conclusions

As requested by the Protecting Our Infants Act of 2015, information presented in this article identifies some of the major gaps in prenatal opioid abuse research such as inconsistencies in treatment success, lack of availability in treatment options, and lack of a consensus on a standard of care. Specifically, this article provides considerations for the three most appropriate opioid maintenance therapy options for pregnant women according to the American College of Obstetricians and Gynecologists. Although each treatment option presents some benefits in reducing opioid abuse and negative outcomes for the fetus, weaknesses and inconsistencies of each present the overarching need for an improved standard of care. To improve prenatal opioid abuse therapy options, drug development must significantly place focus on availability in terms of healthcare coverage and treatment costs, success in retention rates for opioid-abusing patients postpartum, and minimizing negative outcomes for the fetus and newborn regarding the previously stated demographic groups. Future research is therefore pertinent in addressing the aforementioned gaps in order to combat the increasing prevalence of prenatal opioid abuse.

## Abbreviations

B/N: Buprenorphine/naloxone
CNS: Central nervous syndrome
GI: Gastrointestinal
NAS: Neonatal abstinence syndrome
NMDA: N-methyl-D-aspartate
US: United States
